# Supplementary material for: Relationships between Freezing of Gait Severity and Cognitive Deficits in Parkinson’s Disease
Source: Brain Sci. 2021 Nov 12;11(11):1496. doi: 10.3390/brainsci11111496 (PMC8615553; doi:10.3390/brainsci11111496)
Supplement: Supplementary file 1 [file brainsci-11-01496-s001.zip › brainsci-1439391-supplementary.pdf]

Supplementary Materials

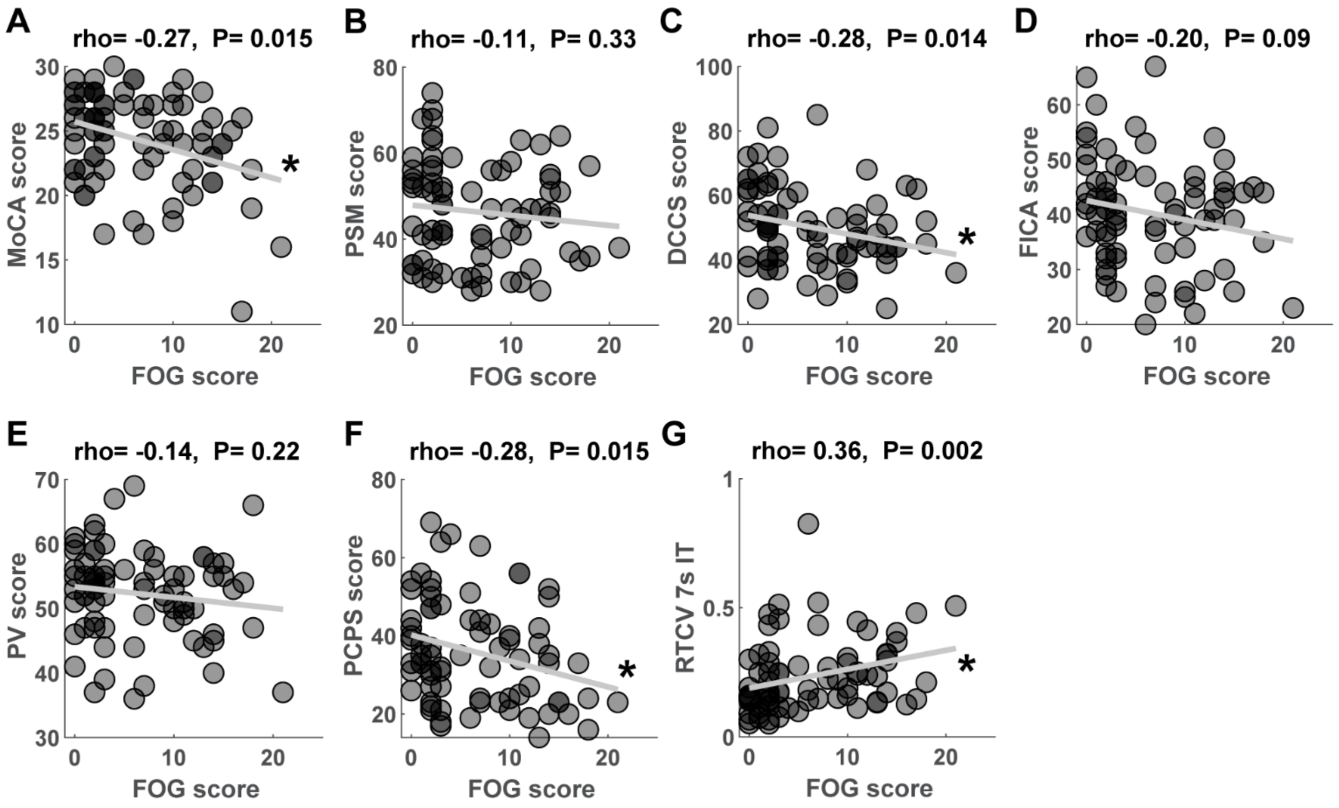

Fig. S1. Spearman correlation plots between FOG scores and cognitive measurement scores (A-G) in PD patients. Higher FOG score denotes severe FOG, lower MoCA score (A) and NIH toolbox cognitive tests (B-F), and higher RTCV (G) denote poor cognitive control. * $p<0.05$ represents significant correlation.

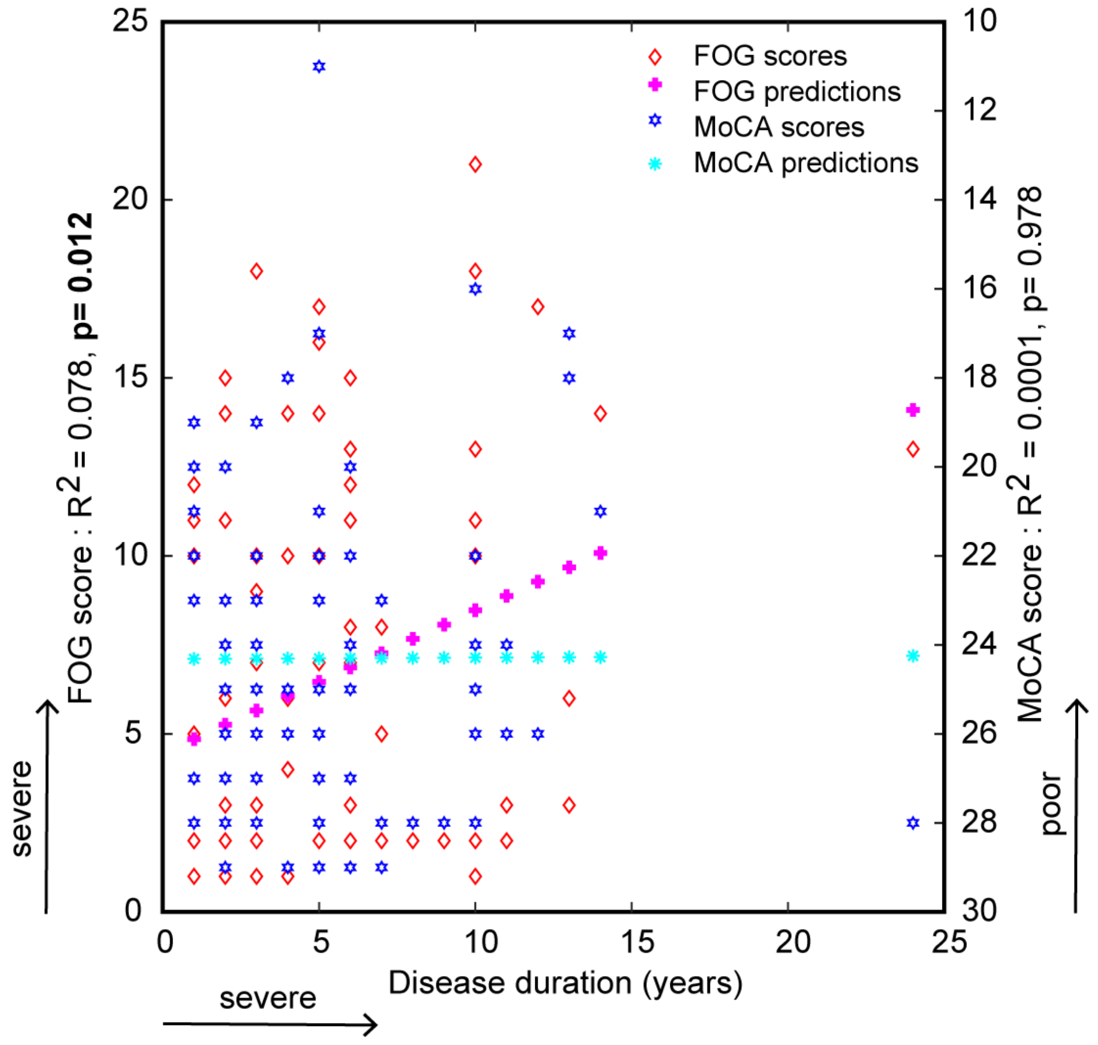

Fig. S2. Linear regression analysis and predicted responses between disease duration and FOG severity and cognitive assessment (MoCA) scores. Bold p-value represents the significance.

Table S1. Cognitive Measurements between PDFOG+ and PDFOG- controlling for variables

	**Model 1**	**Model 2**	**Model 3**	**Model 4**	**Model 5**	**Model 6**	**Model 7**
**Variable**	**Age**	**Gender**	**Years of Education**	**Disease Duration**	**Age, Gender, Education**	**Age, Gender, Education, Disease Duration**	**Age, Gender, Education, Disease Severity**
Cognitive function							
MoCA	4.14 (0.045)*	3.74 (0.057)	5.22 (0.025)*	4.3 (0.041)*	4.55 (0.034)*	4.7 (0.034)*	0.68 (0.411)
Episodic memory							
PSM	0.99 (0.324)	1.66 (0.202)	0.68 (0.413)	1.52 (0.222)	0.049 (0.487)	0.43 (0.515)	0.58 (0.448)
Executive function/Attention							
DCCS	7.96 (0.006)**	8.51 (0.005)**	8.67 (0.004)**	7.9 (0.006)**	7.78 (0.007)**	7.44 (0.008)**	2.03 (0.159)
FICA	2.45 (0.122)	2.66 (0.107)	2.4 (0.126)	2.1 (0.152)	1.64 (0.205)	1.07 (0.304)	0.18 (0.673)
Language							
PV	1.92 (0.17)	1.81 (0.182)	1.6 (0.21)	2.06 (0.155)	1.21 (0.276)	1.28 (0.262)	1.29 (0.259)
Processing speed							
PCPS	1.46 (0.231)	1.9 (0.173)	2.23 (0.14)	2.03 (0.158)	1.46 (0.232)	1.53 (0.22)	0.07 (0.79)
Temporal processing/Working memory							
RTCV	4.9 (0.03)*	4.25 (0.043)*	3.77 (0.056)	3.99 (0.05)*	2.98 (0.089)	2.36 (0.129)	0.50 (0.484)

ANCOVAs for differences in cognition between PDFOG+ and PDFOG- controlling for variables. Model 1, adjusting for age; Model 2, adjusting for gender; Model 3, adjusting for years of education; Model 4, adjusting for disease duration; Model 5, adjusting for age, gender, years of education; Model 6, adjusting for all covariates; Model 7, adjusting for age, gender, years of education, and disease severity. Presented as F-value (p-value). * $p<0.05$; ** $p<0.01$

Abbreviations:

MoCA: Montreal Cognitive Assessment

PSM: Picture Sequence Memory test of the NIHTB-CB

DCCS: Dimensional Change Card Sort test of the NIHTB-CB

FICA: Flanker Inhibitory Control and Attention test of the NIHTB-CB

PV: Picture Vocabulary test of the NIHTB-CB

PCPS: Pattern Comparison Processing Speed test of the NIHTB-CB

RTCV: Response Time Coefficient of Variation of 7-s interval timing task
